# A New Anæsthetic

**Published:** 1867-12

**Authors:** 


### Selections.

A New Anæsthetic.—Dr. L. P. Yandell, Jr., alludes to a new anæsthetic, the properties of which have been lately investigated by Dr. Smith, of London. He has administered *tetrachloride of carbon* with happy results in a variety of cases. It is inhaled, at intervals, to the extent of a drachm, and though the effect is transient, he has seen relief follow its use in headache, dysmenorrhœa, chronic metritis, and hay fever. He has also used it in labor, and found it a safe and valuable means of mitigating the pains, without apparently hindering the natural efforts. In some cases of labor, it induces a quiet sleep, and removes for a time the efforts of exhaustion of the nervous system. In many respects, Dr. Smith deems it preferable to chloroform—quite as safe, pleasanter to inhale, and producing the effect desired in smaller quantities.—*Atlanta Med. and Surg. Journal.*

